# Intraocular Lens Power Calculation After Corneal Refractive Surgery

**DOI:** 10.4103/0974-9233.61219

**Published:** 2010

**Authors:** Vahid Feiz

**Affiliations:** Department of Ophthalmology, UC Davis Medical Center, Sacramento, CA, USA

**Keywords:** LASIK, cataract, intraocular lens calculation, refractive surgery, keratometry

## Abstract

Cataract surgery after corneal refractive surgery can be challenging for the ocular surgeon due to the difficulty with accurate intraocular lens (IOL) power determination and unexpected refractive surprises. As clinicians have done more work, a number of error sources have been determined. Furthermore, an increasing number of methods to avoid these refractive surprises have been proposed. The combination of this work has resulted in recommendations for the modification of standard IOL power calculations to improve outcomes. The following article includes a brief on, and by no means, inclusive, error sources and ways to compensate for them.

## INTRODUCTION

An increasing number of patients undergo corneal surgical procedures to decrease dependence on glasses or contact lense. These procedures alter corneal effective power. Excimer laser keratectomy has quickly become the modality of choice for corneal refractive surgery, replacing older incisional surgeries such as radial keratotomy (RK).^1^,^2^

As surgeons gain experience with cataract extraction in postrefractive surgery patients, they are finding that standard intraocular lens (IOL) formulas and keratometry can lead to “refractive surprises.” The most common observation is underestimation of IOL power and unexpected hyperopia after cataract surgery in patients who have undergone corneal refractive surgery for correction myopia, regardless of the procedure.^3^–^11^ Moreover, these refractive surprises seem to be directly related to the amount of keratectomy performed. Clinically, this means that greater refractive corrections correlate with greater errors of IOL power.^12^–^14^

Experience with IOL power determination after corneal surgery to correct hyperopia remains limited. A few reported cases of cataract surgery after hexagonal keratectomy (now abandoned) resulted in myopic surprises.^15^ As procedures like hyperopic LASIK/PRK have gained wider acceptance, surgeons can expect to encounter different refractive surprises after cataract surgery in this population.

IOL power determination IOL power calculation relies on three measurements: axial length, corneal power and anterior chamber depth, which are not independently measured. An error in any of these three parameters can lead to a possible refractive surprise.

Historically, axial length measurements have been the source of most refractive surprises, although refinements in biometry techniques and instruments have decreased these errors.^16^,^17^ Assuming accurate biometry, axial length measurements are unlikely to contribute significantly to IOL power errors after corneal refractive surgery. Two studies analyzing axial length before and after RK and excimer keratectomy found no significant differences.^18^,^19^

Effective lens position (ELP) or anterior chamber depth affects post-cataract surgery refraction so that a greater myopic shift is observed with more anterior IOL position. Anterior chamber depth cannot be independently measured because even after in-the-bag implantation, it is hard to predict the exact distance between the cornea and the IOL. If corneal surgery significantly changes anterior chamber depth and therefore the ELP, the result can effectively change post-cataract surgery refraction. Several investigators have looked at anterior chamber depth after refractive surgery. One study reported a small forward shift of the posterior cornea after myopic LASIK. This observation, however, has not been confirmed in a similar study.^20^,^21^ These changes, even if real, appear too small to account for changes in refraction and therefore probably do not significantly contribute to IOL power errors after myopic treatments.

Corneal power calculations rely on determining the radius of curvature of the anterior cornea in meters (r), which is converted into a diopteric power (P) using an index of refraction (n) utilizing the following formula.

P=(n-1)/r

Radius of curvature is measured by manual keratometry, automated keratometry or topography. Two assumptions regarding topography or keratometry are that: (1) the cornea is a true spherical surface and (2) the power of the cornea's para-central 3–4 mm is not significantly different from that of the central cornea. These assumptions are clinically acceptable in most normal eyes. In reality, however, the cornea is a prolate, aspheric refractive media with progressive flattening toward the periphery.

## SOURCES OF ERROR IN CORNEAL POWER DETERMINATION

Considering that different types of refractive surgery fundamentally alter corneal shape and power, the usual assumptions no longer apply and may be the sources of error in determining corneal power. In this review of possible error sources, we have divided corneal refractive surgery into RK and excimer keratectomy (PRK, LASIK, LASEK).

### RK

RK steepens the peripheral cornea and flattens the central cornea, resulting in a hyperopic shift and a proportionally greater flattening of the cornea in the center compared with the paracentral cornea.^22^ This creates an abrupt change from treated to untreated cornea. Because keratometry and topography units measure radius of curvature in the cornea's para-central 3–4 mm, the measured diopteric power is significantly steeper than the central cornea. The measured zone also increases in size further from the central cornea as the cornea becomes flatter, resulting in overestimation of cornea power.^23^,^24^

### Myopic excimer keratectomy

The ability of large optical zones to decrease post-operative glare and halos has become evident with increased LASIK and PRK experience, and optical zones >5−6 mm are now considered routine. As a result, the para-central radius of curvature would be expected to closely approximate central corneal curvature. In clinical experience, however, when the radius of curvature is converted to diopteric power, this calculated value overestimates central corneal power.^4^–^12^ This occurs for two main reasons:

First, after excimer keratectomy, the anterior corneal surface changes but the posterior corneal surface remains unaltered. Sonergo-Krone *et al.* found small changes in the posterior corneal power after LASIK but large changes in the anterior–posterior power ratio.^25^ Changing the anterior–posterior power alters the cornea's effective refractive index in direct relation to the amount of keratectomy. In the original Gullstrand model, for every 9% change in ratio, the effective corneal power is changed by 0.5 diopters.^26^

The second factor is the variation in corneal refractive index of the different layers of the cornea. This was shown by Patel *et al*., who found the index of refraction to be slightly different in different layers.^27^ Because excimer laser selectively removes anterior stromal layers and leaves the posterior stroma intact, it changes the cornea's total refractive index. Removing more tissue is also expected to produce a greater change in the refractive index. This is supported by the observed correlation between depth of ablation and error in IOL power after myopic PRK.^12^,^28^

### Hyperopic excimer keratectomy

Little, if any, experience with cataract surgery after hyperopic excimer keratectomy has been reported. Because these treatments cause steepening of the central cornea with large optical zones, para-central radius of curvature, measured by manual keratometry or topography, should be a fairly accurate estimation of central curvature. As in myopic treatments, the anterior–posterior corneal power ratio is expected to change, although in the opposite direction. Therefore, using the standard refractive index would theoretically underestimate corneal power and result in unexpected myopia after IOL implantation.

In our center, we analyzed eight eyes after hyperopic LASIK, using pre-LASIK keratometry and amount of hyperopic treatment to predict a fictitious post-LASIK IOL power. In each case, the predicted IOL power was lower than the IOL power determined by standard post-LASIK keratometry.^13^ Despite a lack of actual implantation, this study indicated that using post-hyperopic LASIK standard keratometry could theoretically result in IOL power overestimation and unexpected myopia.

### Summary

Manual keratometry after myopic L ASIK, PRK and RK overestimates corneal power and underestimates IOL power. The causes differ for RK and LASIK/PRK. In LASIK/PRK, error is directly proportional to the amount of keratectomy. Manual keratometry after hyperopic L ASIK and PRK theoretically underestimates corneal power and results in IOL power overestimation, also in direct proportion to the amount of correction.

## METHODS TO IMPROVE IOL POWER DETERMINATION

Several methods can improve IOL power accuracy after corneal refractive surgey. No single approach has been studied in a large sample, and some are based purely on theory. Most cases also require knowledge of pre-refractive surgery data that may not be available to cataract surgeons. Proposed methods include use of topography to measure central corneal power, advanced IOL calculation formulas, contact lens over-refraction, clinical history, nomogram-based adjustment, corneal power determination by directly determining posterior curvature and intentional overcorrection targeting for myopia.

### Topography

Topography-measured corneal power has been suggested to improve central corneal power measurements in post-refractive surgery eyes. Hussein *et al.* developed the topography method to calculate the corneal power within the pupil.^28^ The study showed that the average central power differed from standard keratometry in post-refractive surgery eyes having small optical zones and large attempted corrections. Theoretically, this method offers advantages in eyes with small optical zones.

By contrast, Seitz *et al.* found manual keratometry to be superior to topography-derived values in post-myopic PRK eyes.^12^,^29^

In summary, using topography to determine central corneal power may be beneficial after RK with small optical zones. However, topography has not been found to be superior to standard keratometry in post-PRK/LASIK corneas, and its reliability and accuracy have not been verified.

### Using advanced formulas

Modern theoretic optical formulas (Holladay, Hoffer Q, SRK-T) may offer improved accuracy of IOL power determination in post-refractive surgery eyes. Koch *et al*.^4^ found the Binkhorst and Holladay formulas to be superior to SRK II in post-RK eyes. Odenthal *et al.* noted that using the Hoffer Q formula after myopic LASIK decreased, but did not eliminate, IOL power underestimation.^30^

Another popular formula proposed by Aramberri, know as the double K method, utilizes pre-refractive surgey Ks to estimate an ELP and post-refractive surgery Ks are used to determine IOL power taking into account the ELP.^31^

A number of other formulas have been proposed by other authors. Some include Haigis-L, Latkany formula, etc. A review of all these is beyond the scope of this article.

Although these studies offer no clear-cut conclusions regarding the accuracy of different modern theoretic formulas, their use is probably advantageous in post-refractive surgery eyes.

### Contact lens over-refraction

This method uses a hard contact lens of known power and base curve to determine true corneal power. After patients have undergone refraction, a plano hard contact lens is placed on the eye and over-refraction is performed. If no difference exists between refractions, corneal power is the same as the contact lens base curve. If over-refraction is more myopic than refraction without the contact lens, the lens is steeper than the cornea. The change in refraction is subtracted from the contact lens base curve to yield corneal power. If over-refraction is more hyperopic than the contact lens refraction, the cornea is steeper than the lens. Change in refraction is added to the contact lens base curve to calculate corneal power.

Contact lens-derived corneal powers have been shown to correlate well with manual keratometry in normal corneas when visual acuity is better than 20/70.^32^ Once the visual acuity is lower than 20/70, which may be the case in many patients with cataract, the correlation is poor. The accuracy of this technique is not established in post-refractive surgery eyes.

### Clinical history

Originally proposed by Holladay to determine corneal power after RK, this method was advocated by Hoffer for use in post LASIK/PRK eyes.^33^,^34^ Using this method requires knowledge of keratometry prior to refractive surgery as well as induced refractive change before the development of cataract. These values are used to determine a calculated corneal power as follows:

For post-myopic (post-RK/myopic excimer) procedures: Corneal diopteric power = pre-refractive surgery Ks – change in SE.

For post-hyperopic (post-hyperopic excimer) procedures: Corneal diopteric power = pre-refractive surgery Ks + change in SE.

The major shortcomings of this approach are that accuracy and reliability have not been established in large series and that it requires knowledge of keratometry values prior to refractive surgery, which cataract surgeons may not have. Its major flaw, however, is assuming a one-to-one relation between corneal diopteric power and refraction (i.e., if corneal power changes by one diopter, refraction changes by one diopter). Studies by Patel *et al.* and Hugger *et al*. analyzed changes in refraction and corneal power after refractive surgery in a large sample.^35^,^36^ Both studies found less change in corneal power than in refraction and concluded that this was due to a change in the cornea's effective refractive index. This indicates that the clinical history method reduces IOL power errors but the degree of accuracy is not yet established.

### Nomogram-based correction

By analyzing eyes after myopic and hyperopic LASIK, we developed a theoretic nomogram to correct IOL power after these procedures.^13^ The nomogram is based on four established clinical premises:

1. IOL power after myopic corneal surgery has to be higher than before surgery.
2. IOL power after hyperopic corneal surgery is expected to be lower than before surgery.
3. To maintain emmetropia, the difference between IOL powers before and after refractive surgery must compensate for refraction changes.
4. For every diopter of change in IOL power, refraction at the spectacle plane with a vertex distance of 12.5 mm changes by only 0.67 diopters.^37^

These formulas allowed the development of a nomogram to adjust IOL power based on post-LASIK standard keratometry [Tables 1 and 2] and eliminated the need for pre-LASIK keratometry. Compared with the clinical history method, this nomogram gave a higher IOL power after myopic LASIK and lower IOL power after hyperopic LASIK.

**Table 1:** Nomogram for intraocular lens (IOL) power adjustment for emmetropia after myopic LASIK

Change in Spherical Equivalent at the Spectacle Plane Induced by myopic LASIK/ Photorefractive Keratectomy (Diopters)	Increase in Intraocular Lens Power (Diopters)
1.00	0.36
1.50	0.66
2.00	0.96
2.50	1.26
3.00	1.55
3.50	1.85
4.00	2.15
4.50	2.45
5.00	2.74
5.50	3.04
6.00	3.34
6.50	3.64
7.00	3.93
7.50	4.23
8.00	4.53
8.50	4.83
9.00	5.12
9.50	5.42
10.00	5.72
10.50	6.02
11.00	6.31
11.50	6.61
12.00	6.91

This table is reprinted with permission from Feiz V, Mannis MJ, Garcia FF, *et al*. Intraocular lens power calculation after laser in situ keratomileusis for myopia and hyperopia. Cornea 2001;20:702–97. The copyright is held by the publisher Lippincott Williams & Wilkins.

**Table 2:** Nomogram for IOL power adjustment for emmetropia after hyperopic LASIK

Change in Spherical Equivalent at the Spectacle Plane Induced by hyperopic LASIK/Photorefractive Keratectomy (Diopters)	Decrease in Intraocular Lens Power (Diopters)
	
1.00	0.00
2.00	0.97
3.00	1.84
4.00	2.70
5.00	3.56
6.00	4.42

This table is reprinted with permission from Feiz V, Mannis MJ, Garcia FF, *et al*. Intraocular lens power calculation after laser in situ keratomileusis for myopia and hyperopia. Cornea 2001;20:702–97. The copyright is held by the publisher Lippincott Williams & Wilkins.

This nomogram has been tested and appears to be reliable in a limited number of studies.^13^ Further prospective data of this method's accuracy are currently being collected.

### Optical formula corneal power calculations

Using Gaussian optics, the cornea's true power can theoretically be determined regardless of previous surgical procedures. Thisapproach considers the cornea to have two refractive surfaces, anterior and posterior. The theoretic power of the cornea is calculated using corneal thickness and refractive indexes of air, cornea and aqueous humor through a series of formulas.

Hamed *et al.* used this method to look at 100 post-myopic LASIK eyes. The authors used a mathematical optical formula to directly calculate corneal refractive power.^38^

Good theoretical correlation was noted between this calculated corneal power and the clinical history method. To our knowledge, no actual IOL implantations based on this formula have been performed.

### Direct corneal power measurements

The major shortcoming with all the above-mentioned techniques is the need to know the pre-refractive surgery values, such as refraction and keratometry. An ideal method would determine corneal power accurately without these values. True corneal power could be determined regardless of the refractive status if anterior and posterior corneal curvatures could be directly measured. However, direct measurement of the posterior curvature was not possible until recently.

Introduction of slit-beam scanning combined with placido-disk topography Orbscan allows posterior power measurements.

This technology also allows analysis of central optical zones as small as 1–2 mm.^39^

Sonego-Krone *et al.* as well as Seitz *et al.* used this technology for post-myopic LASIK, comparing refractive changes at the corneal level induced by LASIK with Orbscan-measured central total powers within the central 2-mm zone.^25^,^40^ They found a good correlation between expected central diopteric power and measured values, and recommended using central 2-mm power measured by Orbscan for IOL power determination after myopic LASIK. Qazi *et al.* also used a similar method for post-myopic LASIK patients with good results.^41^

Although a promising technology, the accuracy and applicability of these power measurements have not been established clinically.

## TARGETING MYOPIA

When regular keratometry is performed after myopic refractive surgery, selective choice of an IOL to target myopia when other data are not available may reduce refractive surprises. In analyzing eyes undergoing cataract surgery after RK, Chen *et al.* found that selecting an IOL targeting –1.50 in post-RK eyes reduced the frequency of post-cataract hyperopia by 60%. Some initial hyperopia immediately after cataract surgery also regresses over several weeks, possibly because of inherent instability of the post-RK cornea.^42^–^44^

## CONCLUSION

Current methods of IOL power determination after corneal refractive surgery are limited by a lack of actual clinical experience on a large scale and by the theoretic nature of all the calculation methods. However, based on accumulated clinical experience, several useful guidelines can be followed.

In addition to the recommendations below, refractive surgeons should consider providing patients with pre-refractive surgery keratometry and refraction and having them keep these records for possible cataract surgery in the future.

1. If only pre-and post-corneal surgery refraction are available, use post-refractive surgery keratometry and axial length and adjust IOL power using a theoretic nomogram [Tables 1 and 2].
2. If pre-refractive surgery keratometry values and refraction are available, predict IOL power theoretically using clinical history or nomogram-based methods. If using the clinical history method, determine changes in spherical equivalent at the spectacle plane rather than the corneal level.
3. If data are not available and patients have visual acuity >20/70, consider the contact lens method.
4. If data are not available and patients have visual acuity <20/70, consider targeting −1.50 to −2.00 for post-myopic refractive surgery patients and +1.00 for post-hyperopic refractive surgery patients.
5. Some hyperopia in the immediate post-cataract surgery can regress in RK patients, so delay intervention through lens exchange or further refractive surgery until the refraction is stable.
6. Inform patients who have had previous corneal refractive surgery of limitations in accurate IOL power calculations. As part of their informed consent for cataract surgery, specifically discuss the possible need for corrective refractive aids, repeat corneal refractive surgery or IOL exchange.
